# A steep-slope transistor based on abrupt electronic phase transition

**DOI:** 10.1038/ncomms8812

**Published:** 2015-08-07

**Authors:** Nikhil Shukla, Arun V. Thathachary, Ashish Agrawal, Hanjong Paik, Ahmedullah Aziz, Darrell G. Schlom, Sumeet Kumar Gupta, Roman Engel-Herbert, Suman Datta

**Affiliations:** 1 Department of Electrical Engineering, Pennsylvania State University, University Park, Pennsylvania 16802, USA; 2 Department of Materials Science and Engineering, Cornell University, Ithaca, New York 14853, USA; 3 Kavli Institute at Cornell for Nanoscale Science, Ithaca, New York 14853, USA; 4 Department of Materials Science and Engineering, Pennsylvania State University, University Park, Pennsylvania 16802, USA

## Abstract

Collective interactions in functional materials can enable novel macroscopic properties like insulator-to-metal transitions. While implementing such materials into field-effect-transistor technology can potentially augment current state-of-the-art devices by providing unique routes to overcome their conventional limits, attempts to harness the insulator-to-metal transition for high-performance transistors have experienced little success. Here, we demonstrate a pathway for harnessing the abrupt resistivity transformation across the insulator-to-metal transition in vanadium dioxide (VO_2_), to design a hybrid-phase-transition field-effect transistor that exhibits gate controlled steep (‘sub-*kT/q*') and reversible switching at room temperature. The transistor design, wherein VO_2_ is implemented in series with the field-effect transistor's source rather than into the channel, exploits negative differential resistance induced across the VO_2_ to create an internal amplifier that facilitates enhanced performance over a conventional field-effect transistor. Our approach enables low-voltage complementary n-type and p-type transistor operation as demonstrated here, and is applicable to other insulator-to-metal transition materials, offering tantalizing possibilities for energy-efficient logic and memory applications.

## Abstract (web summary)

The intrinsic properties of conventional semiconductors limits the speed and efficiency of field-effect transistors. Here, the authors take advantage of the insulator-to-metal transition in vanadium dioxide to create a transistor with reversible and steep-slope switching at room temperature.

## Graphical abstract

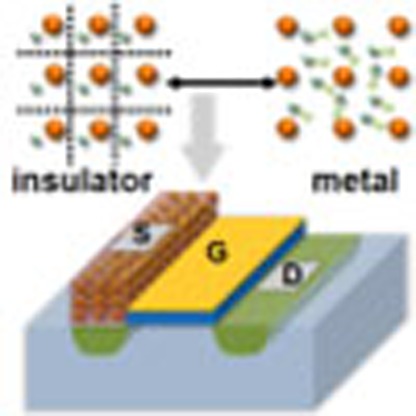

Metal-oxide-semiconductor field-effect transistors (MOSFETs) have been the workhorse of digital computation. In a conventional MOSFET (Fig. 1a), a change in the drain-to-source current (*I*_DS_) can be induced by the application of a transverse electric field across the gate dielectric by means of the third gate terminal. This field lowers the potential energy barrier separating the source and the channel, exponentially increasing the number of carriers traversing the channel. At room temperature, a minimum change of 60 mV in the gate bias (*V*_GS_) is required to effectuate a decade change in *I*_DS_, setting up the so-called ‘60 mV per decade' limit, also known as the ‘Boltzmann limit' (Fig. 1a). Stemming from the statistical distribution of free and independent carriers in conventional semiconductors and determined by the thermal voltage *kT*/*q* (*k*: Boltzmann constant; *T*: temperature; *q*: electron charge), this fundamental limit restricts transistor performance, particularly at low-operating voltages^1^,^2^,^3^,^4^, and has motivated the exploration of FETs that harness collective carrier responses^5^,^6^,^7^,^8^,^9^,^10^,^11^,^12^,^13^. Such collective behaviour—wherein a small external perturbation can trigger an aggregated change in the ground state of the system—can produce internal amplification; and provide a pathway to overcome the Boltzmann limit to enable FET's with sub-*kT*/*q* (*kT*/*ηq*; *η*>1) switching slope and superior performance at low voltages. Particularly, in insulator-to-metal transition (IMT) materials^14^ that exhibit strong correlation, like VO_2_ (refs 15, 16, 17, 18), the collective response to external perturbation (temperature^19^,^20^, pressure^21^,^22^ and electrical stimulus^23^,^24^,^25^,^26^,^27^,^28^) can be the ‘melting' of carriers, marking an electronic phase transformation where the electrons localized at atomic sites change to an itinerant state (Fig. 1b). This phase transformation amplifies the free-carrier concentration^29^; and in the case of VO_2_, manifests itself as a sharp change in resistivity up to five orders in magnitude^30^ at ∼340 K. However, attempts to realize IMT-based three-terminal transistor devices with a solid-state gate dielectric to induce the phase transition directly in the channel material have experienced only limited success^23^,^31^,^32^,^33^. Further, the alternate approach of using an ionic liquid as the gate dielectric, which is the focus of current research^34^,^35^,^36^,^37^,^38^,^39^, is typically slow^40^,^41^,^42^ and susceptible to electrochemical effects^43^. These constraints have restricted the utilization of this collective phenomenon in FETs for advanced high-performance electronic applications.

Here, we explore a novel transistor architecture that harnesses the abrupt free-carrier amplification across the phase transition in VO_2_ using a conventional MOSFET. By electrically coupling the VO_2_ in series with the source of a conventional MOSFET (Fig. 1b), we design a hybrid-phase-transition-FET (hyper-FET) wherein, for a given drain-to-source voltage (*V*_DS_), the gate bias *V*_GS_ modifies the current *I*_DS_ flowing through the MOSFET channel and the VO_2_ in series, triggering an abrupt phase transformation in VO_2_. The proposed hyper-FET not only exhibits steep-slope characteristics but also circumvents the need for a direct field-induced phase transition in VO_2_ with a solid-state gate dielectric. Further, the abrupt resistivity switching of VO_2_ in the hyper-FET configuration, which is the origin of the steep-slope characteristics, induces a negative differential resistance (NDR) across VO_2_ that results in internal voltage amplification which consequently enhances the hyper-FET's performance beyond that of a conventional MOSFET.

## Results

### Experimental demonstration and operation principle

**Figure 2:**
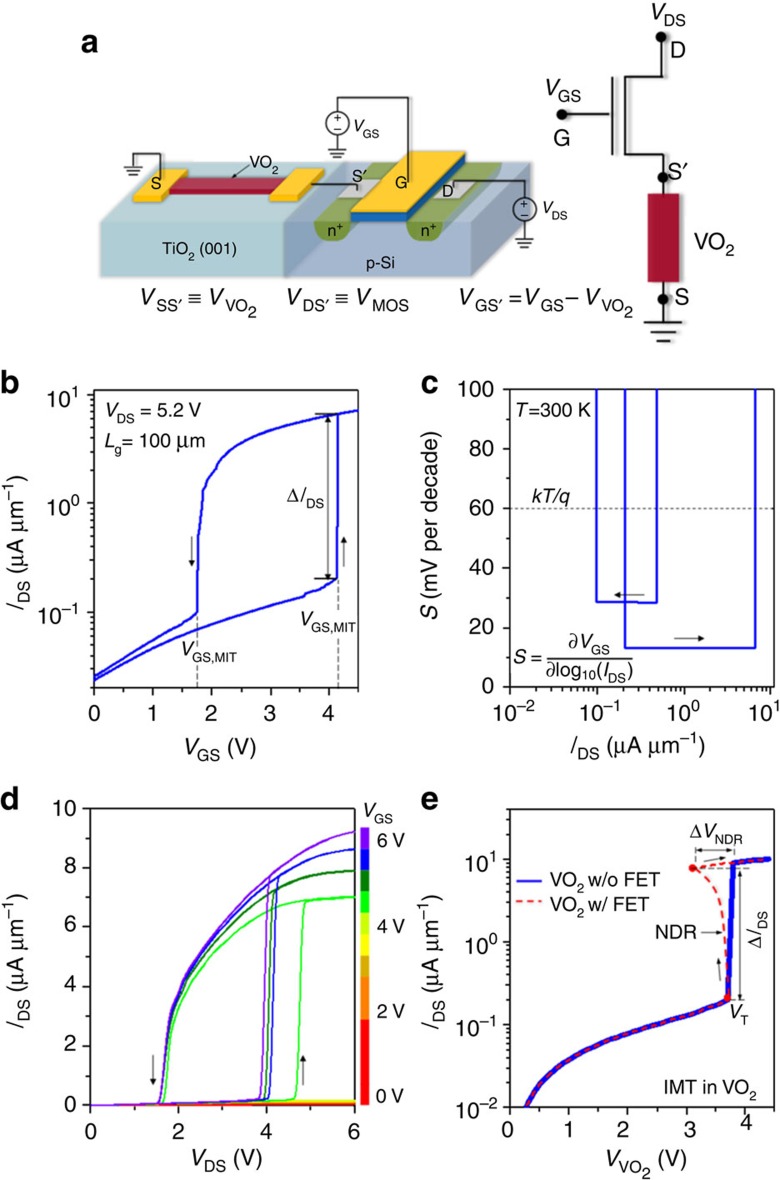
Experimental demonstration of a VO_2_-based hyper-FET. (**a**) Schematic of a hyper-FET consisting of a two-terminal VO_2_ device (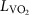
=4 μm; 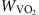
=2 μm) in series with the channel of a conventional Si n-MOSFET (*L*_g_=100 μm; *W*=100 μm). V_VO_2__ is the voltage across the VO_2_ device and *V*_GS′_ is the effective gate-to-source voltage across the MOSFET. (**b**) *I*_DS_–*V*_GS_ transfer characteristics of the hyper-FET exhibiting abrupt and reversible modulation of the channel current *I*_DS_ as a function of the gate-source voltage *V*_GS_. The abrupt turn-ON and turn-OFF of the hyper-FET corresponds to the IMT and MIT in VO_2_, respectively. (**c**) Switching slope (*S*) as a function of *I*_DS_ revealing the steep-slope characteristics (*S*<60 mV per decade) of the hyper-FET during the forward and reverse gate bias sweep. (**d**) Output characteristics (*I*_DS_–*V*_DS_) of the hyper-FET with excellent current saturation. (**e**) Current versus voltage characteristics of the VO_2_ device with (red) and without (blue) the MOSFET in series, illustrating the electrically triggered abrupt IMT. The channel resistance of the MOSFET acts as a series resistor, modifying the current–voltage dynamics through a feedback and inducing a negative differential resistance NDR (red) across the phase transition in VO_2_. The NDR reduces the voltage across the VO_2_ by Δ*V*_NDR_. The current has been normalized to the width of the Si n-MOSFET to show that the abrupt IMT in VO_2_ triggers the abrupt turn-ON of the hyper-FET shown in **b**.

Figure 2a illustrates the schematic of an experimental hyper-FET consisting of a two-terminal VO_2_ device in series with the channel of a conventional Si n-MOSFET (individual device characteristics are shown in Supplementary Fig. 1 and discussed in Supplementary Note 1). All measurements in this work are performed at room temperature (*T*=300 K). The modulation in the transfer characteristics (*I*_DS_–*V*_GS_) of the hyper-FET is shown in Fig. 2b. Initially, at *V*_GS_=0 V (MOSFET in OFF-state), VO_2_ is in the high-resistivity insulating state. In this series combination, *V*_DS_ is divided between the MOSFET channel and the insulating VO_2_ in proportion to their respective resistances; and the current *I*_DS_ through the channel and VO_2_ is insufficient to induce an IMT. As *V*_GS_ increases, the MOSFET-channel resistance decreases until *I*_DS_ reaches a critical current threshold, *I*_IMT_. This triggers an abrupt IMT with the VO_2_ transforming into the low-resistivity metallic state that consequently leads to an abrupt increase in *I*_DS_ (turn-ON). Similarly, as *V*_GS_ reduces, the MOSFET-channel resistance increases until *I*_DS_ drops to a critical threshold value, *I*_MIT_, and the VO_2_ transforms back to the high-resistivity insulating state accompanied by an abrupt reduction in *I*_DS_ (turn-OFF). The difference between the critical threshold values (*I*_MIT_>*I*_IMT_; corresponding to *V*_GS,IMT_, *V*_GS,MIT_, respectively) results in hysteresis (=*V*_GS,IMT_−*V*_GS,MIT_) (Fig. 2b).

Analysing the switching slope 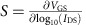, shown in Fig. 2c, it is evident that the abrupt change in current associated with the IMT/MIT in VO_2_ results in steep-slope (*S*<60 mV per decade) characteristics, both during the forward and the reverse *V*_GS_ sweep. We emphasize that the current change Δ*I*_DS_ is abrupt and the extracted value of *S* is limited by the voltage resolution (1 mV). The corresponding output characteristics (*I*_DS_–*V*_DS_) of the hyper-FET (Fig. 2d) show excellent *I*_DS_ saturation behaviour, which is paramount for small signal amplification. This is in contrast to the traditional IMT-based transistor design where the IMT occurs in the channel material^23^. Such a transistor is unlikely to demonstrate current saturation since the design envisages the IMT channel to have a metallic character in the transistor's ON-state which fundamentally cannot sustain a drain side depletion region.

### Internal amplification in the hyper-FET

To elucidate the internal amplification, we analyze the current–voltage dynamics across VO_2_ in the hyper-FET configuration (Fig. 2e). In this series combination, the abrupt IMT results in an NDR across the VO_2_. Such an NDR is induced because when the VO_2_ resistance decreases abruptly, it results in (a) an increase in *I*_DS_ (Δ*I*_DS_) which flows through the VO_2_ device and the MOSFET channel in series; (b) a reduction in the voltage across the VO_2_ device 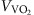 (−Δ*V*_NDR_) (see Supplementary Fig. 2 and Supplementary Note 2 for discussion on the NDR in VO_2_). The effective gate-to-source voltage across the MOSFET (*V*_GS′_; S′: internal node in Fig. 2a) when VO_2_ is in the insulating state (hyper-FET OFF-state) is *V*_GS′_=*V*_GS_−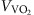. It can be observed that the voltage across the insulating VO_2_ results in an additional voltage drop (=−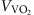) in the effective gate-to-source voltage *V*_GS′_. Across the IMT in VO_2_ which induces the NDR, this voltage drop (=−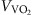) reduces by Δ*V*_NDR_ (therefore increasing *V*_GS′_ by Δ*V*_NDR_; Fig. 2a). Thus, the additional voltage drop (=−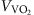) in *V*_GS′_ when VO_2_ is in the insulating state results in a drastic reduction in the OFF-state current (*I*_DS,OFF_) of the hyper-FET in comparison to the stand-alone MOSFET, whereas the reduction in ON-state current (*I*_DS,ON_) is much less significant since the voltage drop across the metallic VO_2_ is small; this results in an overall enhanced current change that is, a higher *I*_DS,ON_/*I*_DS,OFF_ ratio (see Supplementary Fig. 3 and Supplementary Note 3 for additional details and simulations). We model the MOSFET with VO_2_ combination as an equivalent common-source transistor circuit (Fig. 2a) where:


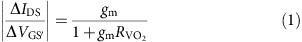


Here, *g*_m_ is the transconductance of the stand-alone MOSFET. Across the IMT, the VO_2_ exhibits an NDR (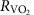
=−Δ*V*_NDR_/Δ*I*_DS_), and therefore equation (1) evolves to:


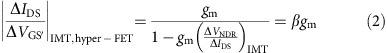


where 
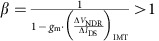


Equation (2) indicates that in a particular gate-voltage window, the amplified differential transconductance (*βg*_m_; *β*>1) of the hyper-FET facilitates a larger change in current compared with the stand-alone MOSFET. The VO_2_, therefore, sets up an internal amplifier (*β*>1) in the hyper-FET, and the transconductance enhancement (*βg*_m_) is directly related to the free-carrier amplification across the phase transition. We note that although the hyper-FET has reduced transconductance before and after the IMT (that is, VO_2_ in the stable insulating/metallic state), the abrupt free-carrier amplification across the IMT overcompensates this reduction and enables an amplified current change. To evaluate the improved performance of the hyper-FET, particularly for digital-logic applications, we match the OFF-state current *I*_DS,OFF_ of the hyper-FET and the stand-alone MOSFET and analyze the increase in ON-state current *I*_DS,ON_ over the operating gate-voltage window, as shown further.

### Low-voltage n-type and p-type hyper-FET operation

**Figure 3:**
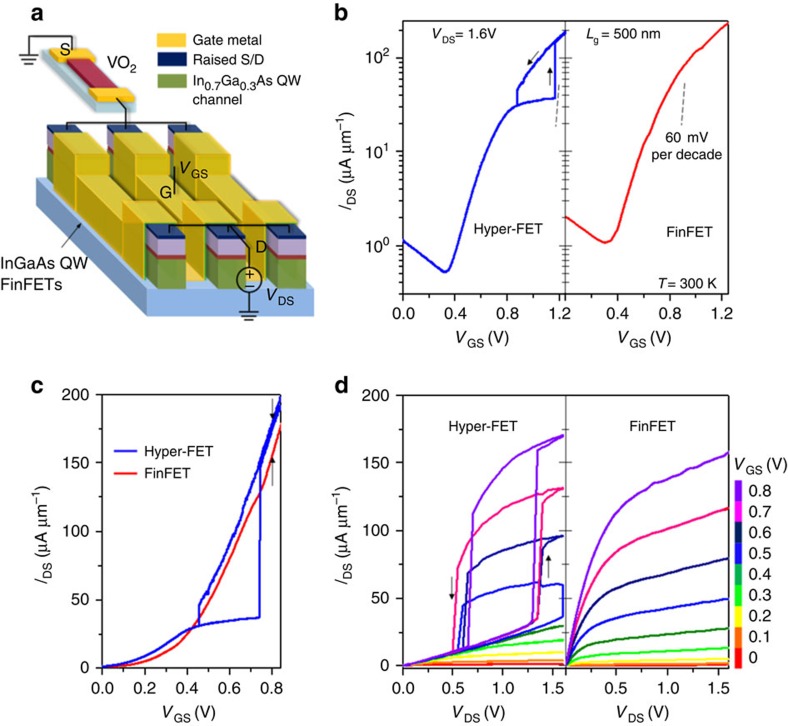
Experimental demonstration a low-voltage hyper-FET using next generation FinFET technology beyond Si. (**a**) Schematic of the n-hyper-FET consisting of a series combination of a scaled VO_2_ (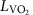
=200 nm) and a multi-channel (=3 fins) In_0.7_Ga_0.3_As quantum-well FinFET (*L*_g_=500 nm). (**b**) Transfer characteristics (*I*_DS_–*V*_GS_) of the hyper-FET and the stand-alone FinFET. (**c**) The positive feedback provided by the VO_2_ enables the hyper-FET to exhibit a ∼20 % higher ON-state current (*I*_DS,ON_) compared with the stand-alone n-FinFET over a gate-voltage window of 0.8 V at matched OFF-state current. (**d**) Output characteristics (*I*_DS_–*V*_DS_) of the n-hyper-FET and the conventional FinFET.

**Figure 4:**
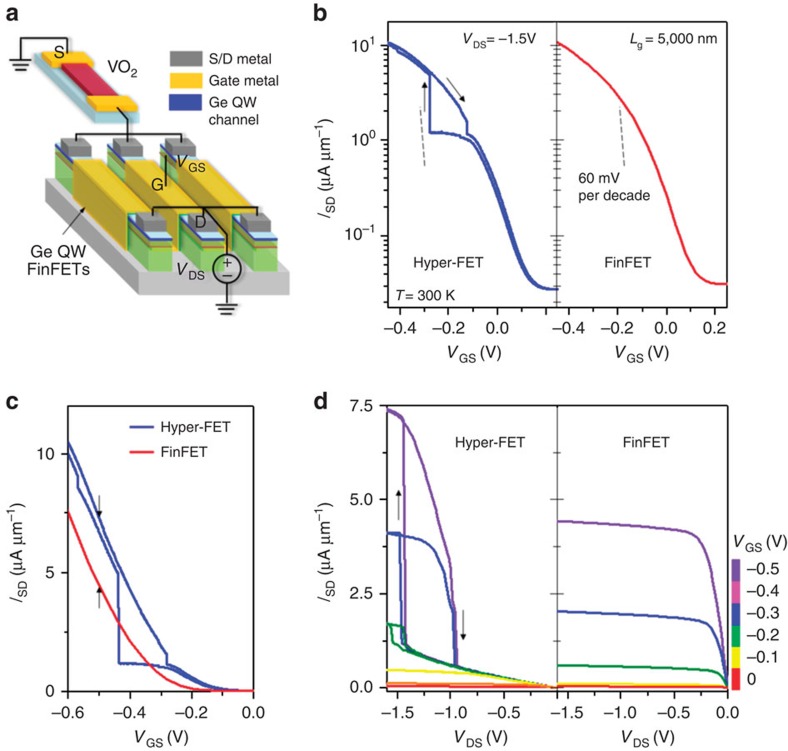
Experimental demonstration of a low-voltage p-type hyper-FET. (**a**) Schematic of the p-hyper-FET consisting of a series combination of scaled VO_2_ (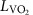
=200 nm) and multi-channel (=200 fins) p-type Ge quantum-well FinFET (*L*_g_=5,000 nm). (**b**) Transfer characteristics (*I*_SD_–*V*_GS_) of the hyper-FET and the FinFET (stand-alone). (**c**) The p-hyper-FET shows a ∼60 % higher ON-state current (*I*_SD,ON_) in comparison to the stand-alone FinFET over a gate-voltage window of −0.5 V at matched OFF-state current. (**d**) Output characteristics (*I*_SD_–*V*_DS_) of the p-hyper-FET and the conventional FinFET.

Next, we focus on the MOSFET component of the hyper-FET. The gate-bias triggers the phase transition in VO_2_ by enabling the MOSFET to source the corresponding critical currents. Therefore, using a scaled transistor can enable low-voltage hyper-FET operation since the transistor can now source the same currents at low *V*_GS_ and *V*_DS_. This motivates the integration of scaled, high-*g*_m_-advanced transistor architectures like FinFETs fabricated on channel materials having mobilities higher than that of silicon to design a low-voltage hyper-FET (Figs 3 and 4).

Figure 3a illustrates a scaled hyper-FET consisting of a scaled In_0.7_Ga_0.3_As quantum-well multi-channel FinFET (*L*_g_=500 nm) (see Supplementary Fig. 4 and Supplementary Note 4 for fabrication method) in series with VO_2_ (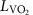
=200 nm; 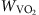
=1 μm). Figure 3b shows the transfer characteristics of the hyper-FET (and the stand-alone FinFET) exhibiting a ‘gate controlled' abrupt turn-ON/turn-OFF associated with the IMT/MIT in VO_2_, respectively. The direct comparison of the hyper-FET with the stand-alone FinFET reveals an improved *I*_DS,ON_/*I*_DS,OFF_ ratio over a *V*_GS_ range of 0.8 V, and thus a ∼20% enhancement in *I*_DS,ON_ at matched *I*_DS,OFF_ (Fig. 3c). The corresponding output characteristics of the hyper-FET and its constituent FinFET, shown in Fig. 3d, also reflect the *I*_DS_ enhancement.

We also demonstrate a p-type hyper-FET since complementary operation, similar to the complementary metal-oxide-semiconductor (CMOS) logic family, is imperative for low standby-power digital applications. Two-terminal VO_2_ devices exhibit reversible switching in both positive and negative voltage polarities (Supplementary Fig. 1) which allows for electrical integration with a p-channel FinFET to enable p-type hyper-FET operation. Figure 4a shows the schematic of a p-hyper-FET constructed using a p-channel Ge quantum-well multi-channel FinFET (see Supplementary Fig. 4 and Supplementary Note 4 for fabrication method) in series with VO_2_ (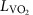
=200 nm; 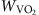
=1 μm). Figure 4b,d shows the transfer characteristics and the corresponding output characteristics of the p-hyper-FET and its constituent FinFET, respectively. The p-hyper-FET also exhibits an enhanced *I*_SD,ON_/*I*_SD,OFF_ ratio over a *V*_GS_ range of −0.5 V, and thus a ∼60% enhancement in *I*_SD,ON_ at matched *I*_SD,OFF_ (Fig. 4c).

## Discussion

The hyper-FET is a device concept that harnesses the phase transition in the IMT material, VO_2_, to enable room temperature, steep-slope, n-type and p-type transistor operation with enhanced performance. These experimental results motivate the realization of a scaled, monolithic hyper-FET design entailing hetero-integration of the IMT material with the conventional FET^44^,^45^,^46^,^47^,^48^. Such an integrated device would have to include careful design considerations for minimizing the device ‘foot-print'^44^, reducing potential self-heating effects as well as ensuring low-contact resistance of both the conventional MOSFET, which can adversely affect its ON-state current (and therefore that of the hyper-FET), and that of the VO_2_, which may possibly affect the magnitude of abrupt current change across the IMT. Further, scaling and optimizing the VO_2_ and the MOSFET properties to enable a scaled hyper-FET device with low OFF-state leakage current relevant to low-power circuit applications^49^, and reduced hysteresis with a complete rail-to-rail swing in a complementary configuration (some of the design considerations are discussed in Supplementary Note 5 and Supplementary Fig. 5) will be key factors in realizing a hyper-FET-based hardware platform that can augment current state-of-the-art technology^49^.

The hyper-FET, demonstrated here, is a manifestation of a design methodology that consolidates the unique properties of phase transition materials like abrupt and reversible resistivity switching, arising from collective carrier dynamics and usually inaccessible in a conventional semiconductor, with the robust field-induced switching dynamics of a conventional MOSFET. Our approach harnesses the abrupt IMT in VO_2_ in the much-desired three-terminal transistor configuration, circumventing the need for direct electric field-induced phase transition. Furthermore, the generality of the hyper-FET design also facilitates this transistor architecture to be extended to other insulator–metal transition systems,^50^,^51^,^52^ thus opening the doors to using electronic phase transition materials in digital applications.

## Additional information

**How to cite this article:** Shukla, N. *et al*. A steep-slope transistor based on abrupt electronic phase transition. *Nat. Commun.* 6:7812 doi: 10.1038/ncomms8812 (2015).

## Supplementary Material

Supplementary Information Supplementary Figures 1-5, Supplementary Table, Supplementary Notes 1-5 and Supplementary References

## Figures and Tables

**Figure 1:**
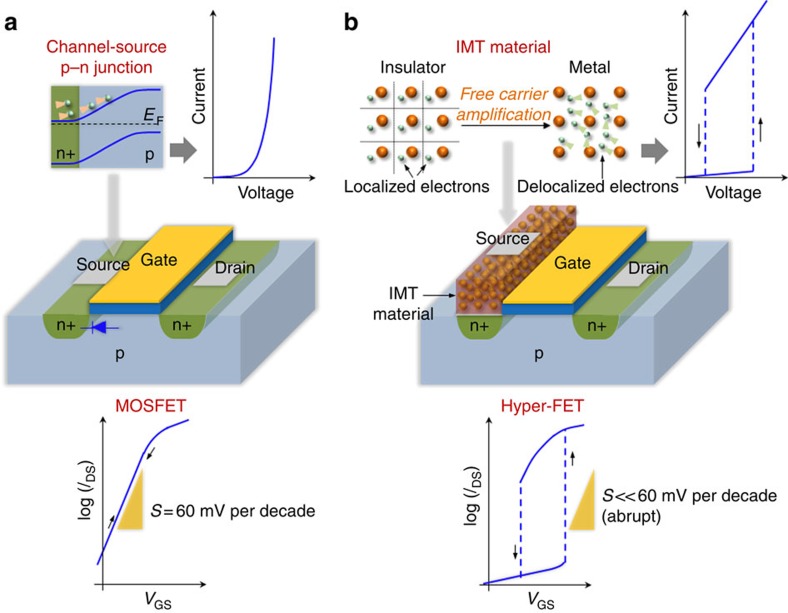
Schematic device design of hyper-FET and working principle. (**a**) Conventional MOSFET and its transfer characteristics (channel current *I*_DS_ versus gate bias *V*_GS_) for a fixed drain-to-source voltage *V*_DS_. The channel-source p–n junction, modulated by the gate terminal, controls the injection of carriers into the channel limiting the switching slope (*S*) of the MOSFET to 60 mV per decade (Boltzmann limit). (**b**) Proposed hyper-FET in which an insulator-to-metal transition (IMT) material that shows electrically induced abrupt resistivity switching is electrically integrated in series with the source of a conventional MOSFET. For a given *V*_DS_, the gate-terminal voltage *V*_GS_ modifies the current flowing through the MOSFET and the IMT material in series, triggering an abrupt phase transition. The associated delocalization of localized carriers (free-carrier amplification) across the IMT results in an abrupt decrease in the resistance of the source, enhancing the switching slope characteristics beyond the intrinsic limits of a conventional p–n junction.
