# Supplementary material for: A steep-slope transistor based on abrupt electronic phase transition
Source: Nat Commun. 2015 Aug 7;6:7812. doi: 10.1038/ncomms8812 (PMC4918311; doi:10.1038/ncomms8812)
Supplement: Supplementary Information — Supplementary Figures 1-5, Supplementary Table, Supplementary Notes 1-5 and Supplementary References [file ncomms8812-s1.pdf]

Supplementary Figures

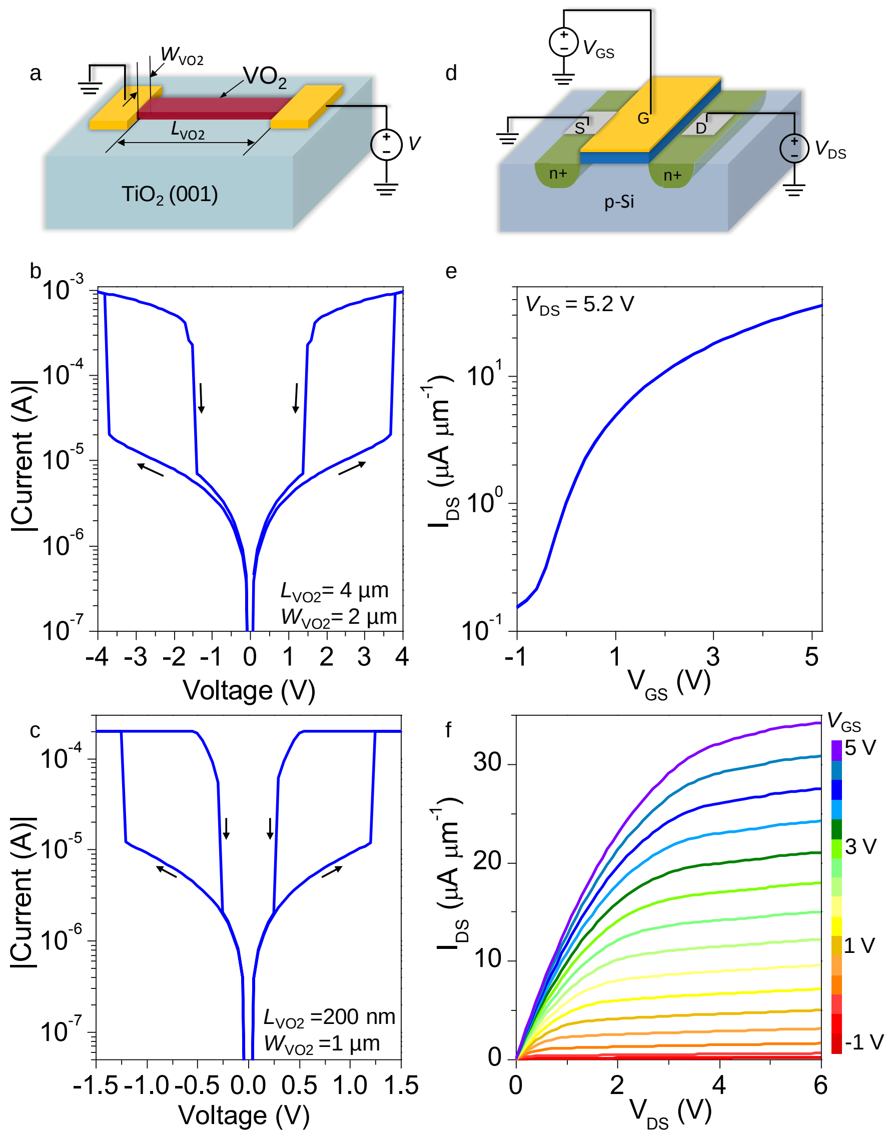

Supplementary Figure 1| Electrical characteristics of stand-alone VO_2 devices and Si n-MOSFET. (**a**) Schematic of a two-terminal VO_2 device. VO_2 is epitaxially grown on a (001) TiO_2 substrate. (**b**) Current versus voltage characteristics of a large two-terminal VO_2 device (L_{VO2}=4 µm; W_{VO2}=2 µm) illustrating the electrically triggered abrupt, hysteretic and reversible resistivity switching in both positive and negative voltage polarities. (**c**) Current versus voltage characteristics of the small two-terminal VO_2 device (L_{VO2}=200 nm; W_{VO2}=1 µm) illustrating that the IMT and MIT are induced at lower voltages. A current compliance is used in the low-resistivity metallic-state to limit the current and prevent thermal runaway in the VO_2 device. (**d**) Schematic of the stand-alone Si n-MOSFET with channel length L_g= 100 µm and width W= 100 µm. (**e**) Transfer characteristics I_{DS}-V_{GS} of the Si n-MOSFET. (**f**) Output characteristics I_{DS}-V_{DS} of the Si n-MOSFET.

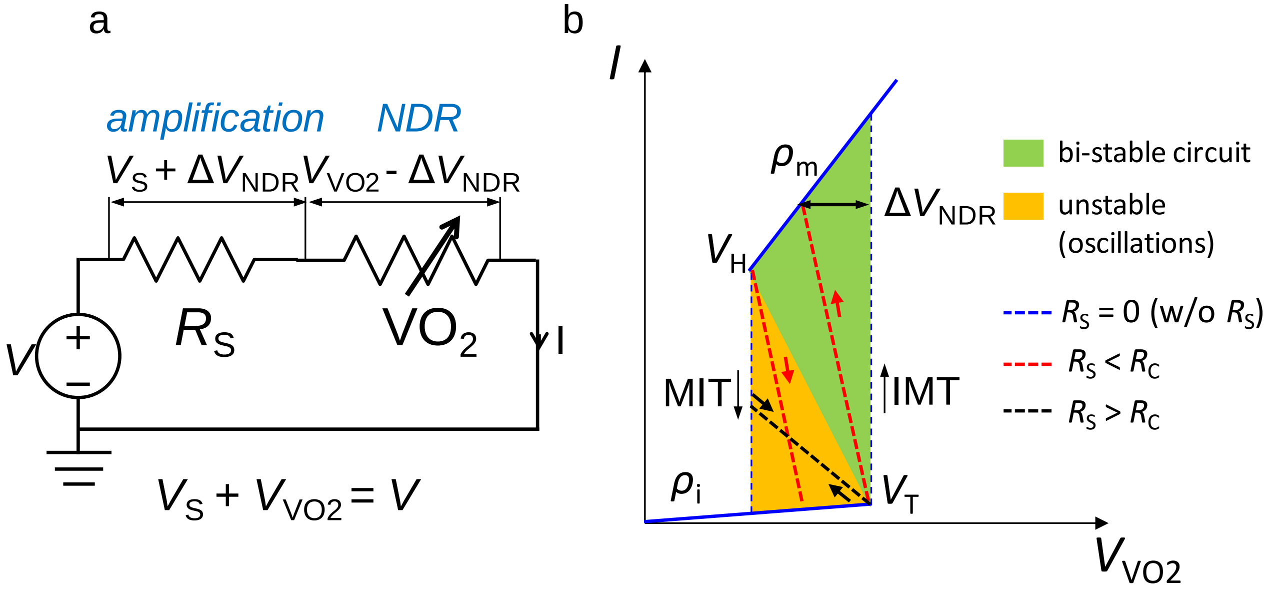

Supplementary Figure 2| Negative-differential-resistance across the insulator-to-metal phase transition in VO_2. (**a**) Circuit schematic of the IMT material, VO_2, in series with a resistor R_S. In case of the Hyper-FET, the channel of the MOSFET is the series resistor R_S (V_{GS}). V_S and V_{VO2} is the voltage drop across R_S and the VO_2 device, respectively. Across the phase transition in VO_2, an NDR results in a voltage drop (= $-\Delta V_{NDR}$) across the VO_2 device (= $V_{VO2} - \Delta V_{NDR}$) with consequent amplification across the series resistor R_S (= $V_S + \Delta V_{NDR}$). (**b**) Schematic of the current-voltage characteristics of a two-terminal VO_2 device without R_S (blue). Addition of a series resistor modifies the current-voltage dynamics across the phase transition in VO_2 (red, black load-lines) enabling the circuit to function in the bi-stable circuit mode (green region), relevant to the Hyper-FET operation or the unstable (oscillatory) mode (yellow region), depending on the value of R_S. R_C is the maximum critical resistance for bi-stable circuit operation.

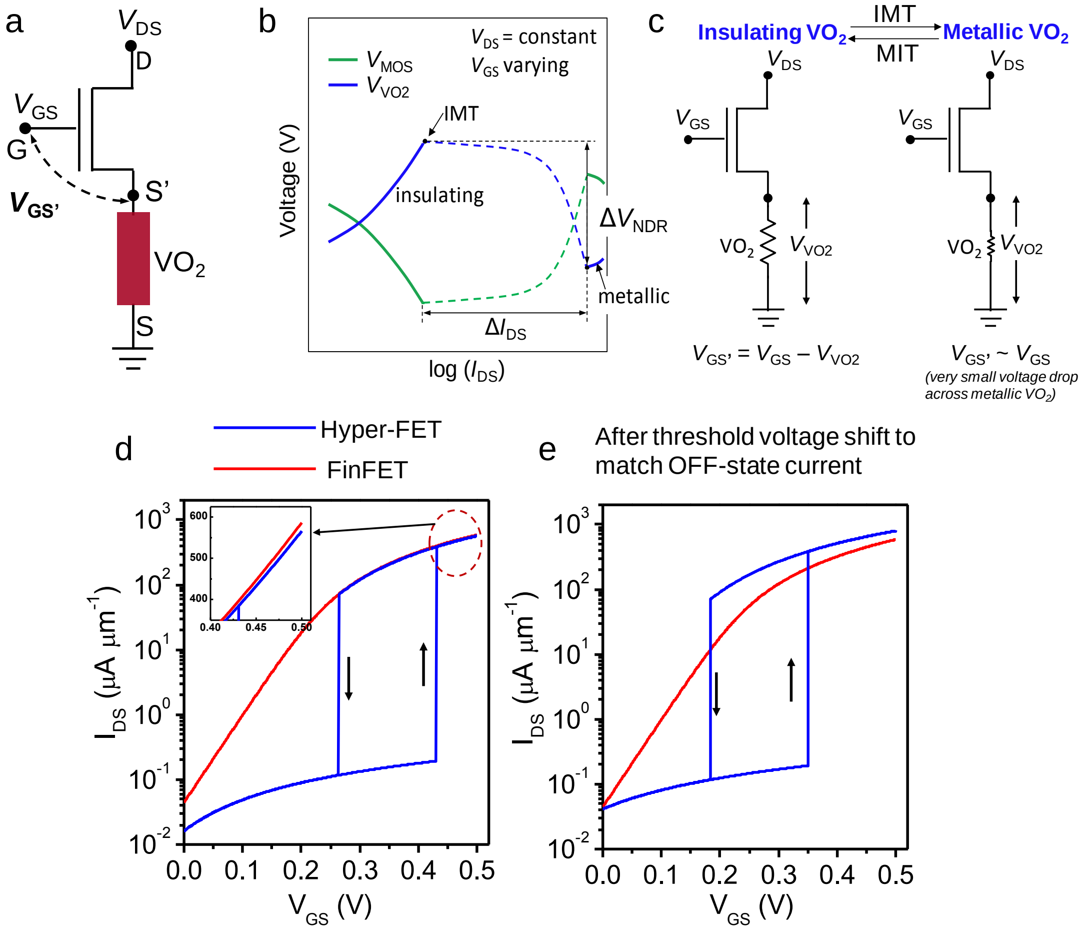

Supplementary Figure 3| Amplification through internal redistribution of voltage within the Hyper-FET. (**a**) Schematic of the Hyper-FET circuit consisting of the VO_2 device in series with the source of the conventional transistor. The corresponding voltages across the various nodes are also shown in the figure. **(b)** Schematic of the redistribution of V_{DS} (constant) between the VO_2 device (= V_{VO2}) and the channel of the conventional transistor (= V_{MOS}) during the Hyper-FET turn-ON (abrupt IMT in VO_2) as a function of channel current I_{DS}. The NDR induced across the VO_2 amplifies the voltage across the transistor channel. (**c)** Voltage redistribution between the conventional transistor and the VO_2 before and after the IMT. Across the IMT, the abrupt snap-back in voltage across the VO_2 also increases the effective gate-source voltage across the transistor $V_{GS'}$ **(d)** Simulated transfer characteristics of the Hyper-FET consisting of a scaled 14nm technology node FinFET (single-fin with fin-height=23 nm, fin-width=10 nm, gate length=18 nm) in series with an IMT material (L=8 nm; W=14 nm; t=14 nm). The Hyper-FET causes a 3.5% reduction in the ON-state current $I_{DS,ON}$ and a 63% reduction in the OFF-state current $I_{DS,OFF}$ in comparison to the stand-alone FinFET resulting in a higher $I_{DS,ON}/I_{DS,OFF}$ ratio. (Inset) shows the magnified image of the ON-state characteristics. **(e)** The OFF-state current of the stand-alone FinFET and the Hyper-FET are matched by shifting the threshold voltage; the net outcome is that the Hyper-FET (after threshold-voltage re-targeting to match the OFF-state current) results in higher ON-state current in comparison to the FinFET.

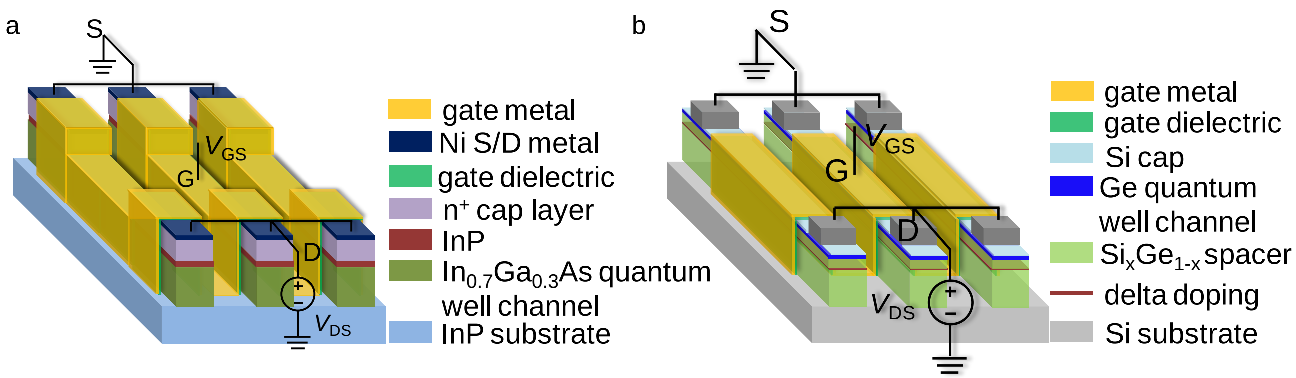

Supplementary Figure 4| Scaled transistor architecture (a) Schematic of the n-$In_{0.7}Ga_{0.3}As$ quantum-well FinFET fabricated on an InP substrate. (**b)** Schematic of a FinFET fabricated on 1.3% strained p-Ge quantum well heterostructure grown on a Si substrate. The Boron delta doping in the SiGe buffer provides free carriers (holes) in the quantum-well channel. Additionally, there is a phosphorous doping in the SiGe buffer to reduce parallel conduction.

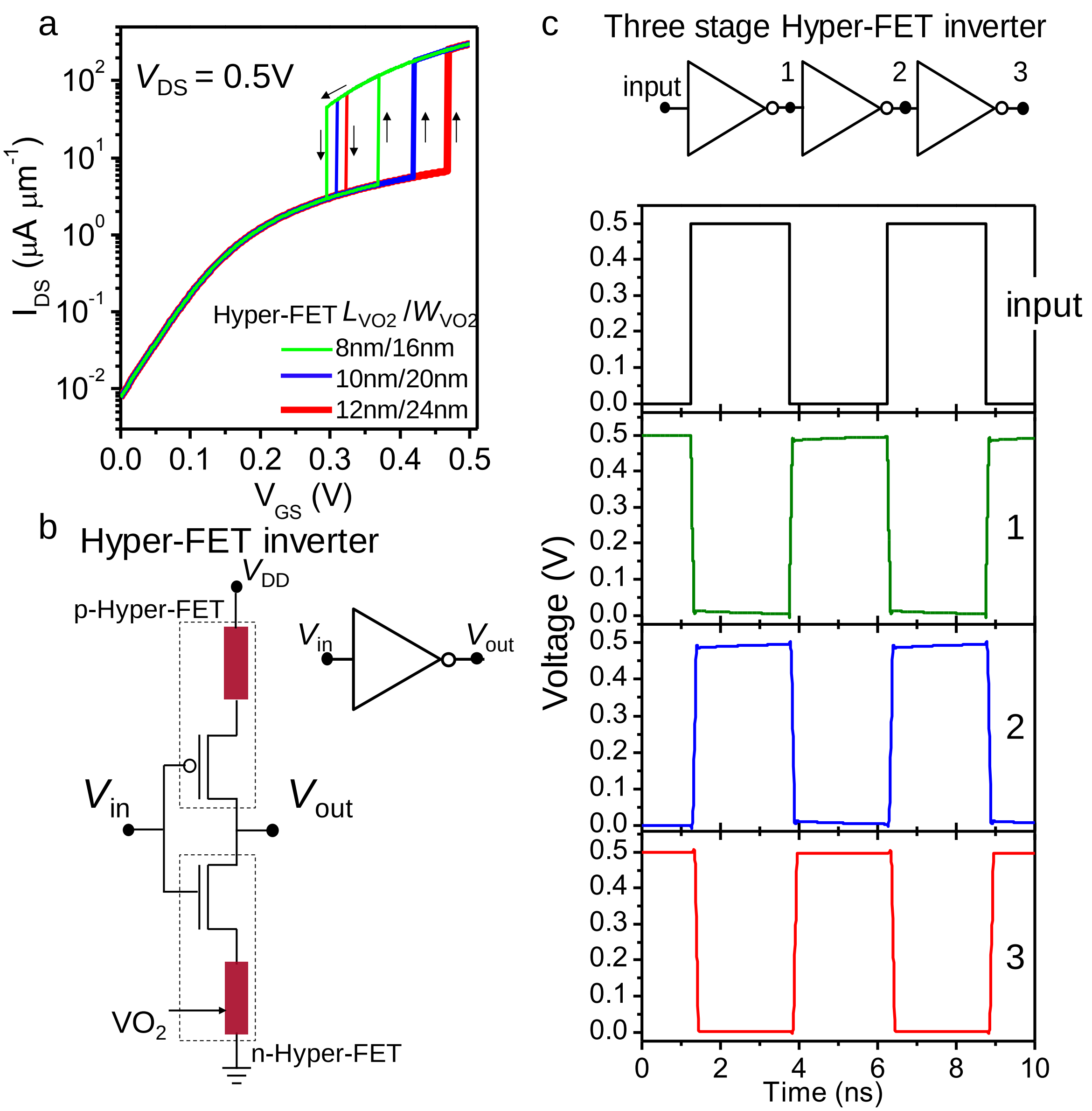

Supplementary Figure 5| Simulation of a scaled Hyer-FET. (a) Simulated transfer characteristics of a Hyper-FET consisting of a 22nm technology node FinFET (single fin with fin-height=28 nm, and fin-width=15 nm) in series with VO_2 of different channel dimensions resulting in different hysteresis. **(b)** Circuit schematic of a Hyper-FET based inverter. **(c)** Schematic of a three-stage Hyper-FET based inverter and its transient response at each node. The output of the third stage is connected to a load capacitor.

Supplementary Table

IMT material parameters	Value
Thickness	20 nm
ρ_m (metallic state resistivity)	10^{-4} Ω.cm
ρ_i (insulating state resistivity)	2 Ω.cm
J_{MIT} (critical current density for MIT)	10^6 (A/cm^2)
J_{IMT} (critical current density for IMT) [Ref. 1]	10^5 (A/cm^2)
C_{VO2}	1 fF

Supplementary Table 1| Parameters for VO_2 used in simulations in Supplementary Note 5

Supplementary Notes

Supplementary Note 1

Electrically characteristics of stand-alone VO_2 and Si n-MOSFET

Two-terminal VO_2 device fabrication. The 10nm thick VO_2 films are epitaxially grown on a (001) TiO_2 substrate employing reactive oxide molecular beam epitaxy using a Veeco GEN10 system. Due to epitaxial mismatch, the VO_2 films are biaxially strained in compression by -0.9%. For the small two-terminal VO_2 devices with L_{VO2}= 200 nm; W_{VO2} = 1 µm, the fabrication starts with patterning the electrodes using electron-beam lithography followed by electron beam evaporation of Ni/Au (15 nm/80 nm) and lift-off in RemoverPG at 343 K. Next, the channel width and isolation are defined by electron beam lithography, followed by a CF_4 plasma based dry etch. Finally, the resist is stripped with RemoverPG at 343 K to reveal the completed devices. For the larger VO_2 devices (L_{VO2}= 4 µm; W_{VO2} = 2 µm), the electrodes are patterned using contact lithography.

Electrically induced insulator-to-metal transition (IMT) in two-terminal VO_2 devices. Supplementary Figure 1a shows the schematic of a two-terminal device fabricated on VO_2 grown on a (001) TiO_2 substrate. Supplementary Figure 1b,c illustrates the electrically triggered, abrupt and

reversible (i.e. metal to insulator transition; MIT) insulator to metal transition (IMT) in both positive and negative voltage polarities for the large (L_{VO2}= 4 µm; W_{VO2} = 2 µm) and the small (L_{VO2}= 200 nm; W_{VO2} = 1 µm) VO_2 devices, respectively. Scaling the VO_2 channel length facilitates the IMT and MIT in VO_2 at lower voltages. To demonstrate the Hyper-FET, the large VO_2 devices (L_{VO2}= 4 µm; W_{VO2} = 2 µm) were electrically integrated with the Si n-MOSFET while the small VO_2 devices (L_{VO2}= 200 nm; W_{VO2}= 1 µm) were electrically integrated with the n^+ source of the n-InGaAs quantum-well FinFET and the p^+ source of the p-Ge quantum-well FinFET, respectively.

Si n-MOSFET electrical characteristics. The transfer characteristics (I_{DS}-V_{GS}) and the output characteristics (I_{DS}-V_{DS}) of the stand-alone Si n-MOSFET are shown in Supplementary Figure 1e,f, respectively.

Supplementary Note 2

Negative-differential-resistance (NDR) and voltage amplification using the electrically induced abrupt insulator-metal transition in VO_2

We evaluate the modification of the electrically induced abrupt resistivity switching dynamics in VO_2 by a series resistor. The current-voltage dynamics of a circuit consisting of VO_2 in series with a resistor R_S can be deduced using simple Kirchoff's circuit laws:

$$\frac{\mathrm{d}V_{\mathrm{VO2}}}{\mathrm{d}V} = \left(1 + R_{\mathrm{S}}\frac{\mathrm{d}I}{\mathrm{d}V_{\mathrm{VO2}}}\right)^{-1} \quad (2.1)$$

where V_{VO2} is the voltage drop across the VO_2 device.

Voltage amplification across R_S is attained when $\frac{\mathrm{d}V_{\mathrm{VO2}}}{\mathrm{d}V} \leq 0$ (i.e. $\mathrm{d}V_{\mathrm{VO2}}$reduces (< 0) as d$V$ increases or remains constant (≥ 0)). VO_2 exhibits an increase in current associated with the abrupt decrease in resistivity (Supplementary Figure 2b). The term 'abrupt' signifies that once the IMT is electrically

triggered, no further external stimulus is required (i.e. ΔV = 0 in a voltage driven measurement) and the VO_2 transitions into the metallic state with no intermediate stable states as indicated by the blue dotted lines in Supplementary Figure 2b. Therefore, $\left(\frac{\mathrm{d}V_{\mathrm{VO2}}}{\mathrm{d}V}\right)^{-1} = 0$ across the IMT; and a circuit with VO_2 in series with a resistor can facilitate voltage amplification across R_S, which is the channel resistance of a MOSFET in case of the Hyper-FET. Substituting ΔV = 0 in Supplementary Equation 2.1,

$$-\mathrm{d}V_{\mathrm{VO2}} = R_{\mathrm{S}}.\mathrm{d}I = \mathrm{d}V_{\mathrm{S}} \equiv \Delta V_{\mathrm{NDR}} \quad (2.2)$$

shows that the voltage 'snap-back' ($-\mathrm{d}V_{\mathrm{VO2}} = -\Delta V_{\mathrm{NDR}}$) across the IMT results in voltage amplification ($\mathrm{d}V_s = \Delta V_{\mathrm{NDR}}$) across the constant resistor (Supplementary Figure 2a). Re-arranging Supplementary Equation 2.2 yields:

$$\frac{\mathrm{d}I}{\mathrm{d}V_{\mathrm{VO2}}} = -\frac{1}{R_{\mathrm{S}}} \quad (2.3)$$

which reveals that the load-line representing the NDR has a slope equal to $-1/R_S$ (Supplementary Figure 2b). This observation has been experimentally verified for VO_2 [2]. In the Hyper-FET, the VO_2 is in series with the channel resistance of a MOSFET which is a gate-voltage controlled non-linear resistor, R_S (V_{GS}). Therefore, the load-line representing the inverse NDR across the IMT in VO_2 is also non-linear as illustrated in Figure 2e of the main text.

Factors controlling the voltage snap-back ΔV_{NDR}. We analyze the factors that control the voltage snap-back ΔV_{NDR} across the IMT. The voltage snap-back associated with the NDR is calculated to be:

$$\Delta V_{\mathrm{NDR}} = (-R_{\mathrm{S}})_{\mathrm{NDR}}.\Delta I = A.(-R_{\mathrm{S}})_{\mathrm{NDR}}.V_{\mathrm{T}}.\left(\frac{1}{\frac{A.R_{\mathrm{S}}}{\rho_{\mathrm{m}}}-1}\right)/\rho_{\mathrm{i}} \quad (2.4)$$

where $A = \frac{W_{VO2}.t_{VO2}}{L_{VO2}}$ is a geometrical area factor. ρ_i, ρ_m and V_T is the insulating-state resistivity, metallic-state resistivity and the trigger-voltage (for IMT) of the VO_2, respectively (Supplementary Figure 2b). ΔI is the change in current between the insulating and the metallic state. Supplementary Equation 2.4 indicates that ΔV_{NDR} depends on the series resistance R_S as well as the properties of the phase transition material (V_T, ρ_m, ρ_i); and for a given change in current (ΔI) across the IMT, a larger R_S results in larger ΔV_{NDR} (larger amplification).

Circuit stability conditions for the Hyper-FET. To achieve bi-stable circuit operation relevant to the Hyper-FET, R_S must be smaller than a critical value R_C given by:

$$R_C = \frac{1}{A} \cdot \frac{V_T - V_H}{\frac{V_T}{\rho_i} - \frac{V_H}{\rho_m}} \quad (2.5)$$

to prevent circuit instability resulting in electrical oscillations[2]. V_H is the hold-voltage for the VO_2 device as shown in Supplementary Figure 2b. $R_S < R_C$ ensures that the load-line corresponding to the IMT and represented by the red dotted line in Supplementary Figure 2b lies in the green region. This load-line facilitates bi-stable circuit operation because it traverses between the stable insulating and the metallic states. When $R_S > R_C$, the circuit load-line (black dotted line in Supplementary Figure 2b) lies in the yellow region and traverses between the unstable states corresponding to the IMT and the MIT (with no hysteresis) which can result in oscillations[2]. In case of the Hyper-FET, where the non-linear channel resistance of a MOSFET is the series resistor, the exact magnitude of ΔV_{NDR} and the circuit stability conditions must be computed numerically.

Supplementary Note 3

Gate bias controlled internal voltage redistribution between the VO_2 and the conventional transistor within the Hyper-FET

Supplementary Figure 3a shows a circuit schematic of the Hyper-FET along with the various nodes and the corresponding voltages across them. Supplementary Figure 3b shows the schematic of the distribution of the total drain-to-source voltage V_{DS} between the channel of the conventional transistor (V_{MOS}) and the VO_2 (V_{VO2}) as a function of I_{DS}. Initially, at V_{GS}= 0 V, the MOSFET is in the OFF-state while the VO_2 is in the high-resistivity insulating-state. As V_{GS} increases, the channel resistance of the transistor decreases, reducing V_{MOS} and subsequently increasing I_{DS}. When this current is sufficient to trigger the IMT, the abrupt decrease in resistivity of the VO_2 results in an abrupt increase in the current (ΔI_{DS}). VO_2 also exhibits an NDR (supplementary Note 2 and main text) resulting in a voltage snap-back across the VO_2. This amplifies the voltage across the transistor channel V_{MOS} (Supplementary Figure 3b), and also results in an increase in the effective gate voltage V_{GS}' (Supplementary Figure 3a) as discussed further.

Comparison of $I_{DS,ON}/I_{DS,OFF}$ ratio of the Hyper-FET and that of the stand-alone conventional transistor within a operating voltage window

In the OFF-state of the Hyper-FET, the insulating nature of VO_2 results in a voltage drop across the VO_2, which reduces the effective gate-source voltage ($V_{GS'}$) across the conventional within the Hyper-FET. Thus, $V_{GS'} = V_{GS}-V_{VO2}$ (Supplementary Figure 3c) instead of $V_{GS'} = V_{GS}$ if the VO_2 was absent; this additional voltage drop results in an exponential reduction in OFF-state current $I_{DS,OFF}$ in comparison to the stand-alone conventional transistor. In the ON-state of the Hyper-FET when VO_2 is in the metallic state, the voltage drop across the *metallic* VO_2 is very small resulting in a small

reduction in the ON-state current of the Hyper-FET in comparison to the conventional transistor. Thus, the small reduction in the ON-state current ($I_{DS,ON}$), and a large reduction in the OFF-state current ($I_{DS,OFF}$) results in a larger $I_{DS,ON}/I_{DS,OFF}$ ratio for the Hyper-FET in comparison to the conventional transistor. To illustrate this at a scaled technology node, we simulate the transfer characteristics (I_{DS}-V_{GS}) of a Hyper-FET constructed from a 14 nm FinFET device[5] [single-fin with fin-height=23 nm and fin-width=10 nm and gate-length=18 nm] in series with a IMT device (L=8 nm; W=14nm; t=14 nm; ρ_i=80 Ω.cm; ρ_m =5x10^{-4} Ω.cm; J_{IMT}=0.55x10^{4} A/cm^{2}, J_{MIT}=2x10^{6} A/cm^{2}), as shown in Supplementary Figure 3d. It is observed that there is a 3.5% reduction in the ON-state current ($I_{DS,ON}$) of the Hyper-FET while the OFF-state current ($I_{DS,OFF}$) reduces by 63.3% resulting in a ~2.64X higher $I_{DS,ON}/I_{DS,OFF}$ ratio in comparison to the stand-alone FinFET.

To compare the performance of the FinFET and the Hyper-FET, we shift the threshold voltage to match the OFF-state current ($I_{DS,OFF}$) of the both devices; and analyze the improvement in the ON-state current $I_{DS,ON}$ of the Hyper-FET, enabled by the higher $I_{DS,ON}/I_{DS,OFF}$ ratio of the Hyper-FET. The transfer-characteristics of the Hyper-FET and the stand-alone FinFET after matching the OFF-state current ($I_{DS,OFF}$), as shown in Supplementary Figure 3e, reveal that the Hyper-FET can exhibit a ~35.85% higher ON-state current in comparison to the stand-alone FinFET.

Supplementary Note 4

Fabrication of n-type $In_{0.7}Ga_{0.3}As$ quantum-well FinFETs and p-type Ge quantum-well FinFETs

$In_{0.7}Ga_{0.3}As$ quantum-well FinFET fabrication. The heterostructure, consisting of a 10nm thick $In_{0.7}Ga_{0.3}As$ quantum-well, is grown on semi-insulating InP using molecular beam epitaxy. Devices are fabricated by first performing a citric acid/H_2O_2 solution based recess etch of the heavily doped $In_{0.53}Ga_{0.47}As$ cap layer to define the gate recess. A well-controlled over-etch is included to remove the 2nm InP etch-stop layer allowing the formation of the gate stack directly on the $In_{0.7}Ga_{0.3}As$ channel. This is followed by the formation of fins within the recessed region. The fin patterns are defined using ZEP 520A electron beam resist. The pattern is first transferred onto an atomic layer deposited (ALD) Al_2O_3 hard-mask through a dry etch step. Subsequently, the fin etch is performed using a Cl_2/N_2 plasma based dry etch. Source/drain metallization is then carried out using a lift-off process with electron beam evaporated Ni. Subsequently the gate stack is formed by first growing an ultrathin passivation layer using alternating cycles of nitrogen plasma exposure with Tri-methyl Aluminium (TMA) pre-pulsing followed by thermal ALD growth of 1nm Al_2O_3 and 2.5nm HfO_2. This is followed by thermal evaporation and lift-off of Ni gate electrodes patterned using electron beam lithography. A schematic of the n-$In_{0.7}Ga_{0.3}As$ quantum-well FinFET[3] is shown in Supplementary Figure 4a.

Ge quantum-well FinFET fabrication. The heterostructure consisting of a 10nm thick Ge quantum well is grown on a Si substrate using Rapid-Thermal-CVD (RTCVD). Devices are fabricated by first performing a pre-clean followed by patterning of the source/drain contact. This is followed by the formation of fins within the recessed region. The fin patterns are defined using ZEP 520A electron beam resist. The pattern is first transferred onto an atomic layer deposited (ALD) Al_2O_3 hard mask through a dry etch step. The fin etch is then performed using a Cl_2/Ar plasma based dry etch. Subsequently, the gate stack is developed. This process includes a. in-situ hydrogen plasma clean; b.

Oxygen-plasma high quality 6Å GeO_x formation; c. 5 Å Al_2O_3 / 35 Å HfO_2 at 250°C. This is followed by e-beam evaporation and lift-off of Ti/Au gate electrodes patterned using electron beam lithography. The gate stack was annealed at 300°C for 10 minutes in forming gas. A schematic of the p-Ge quantum-well FinFET[4] is shown in Supplementary Figure 4b.

Supplementary Note 5

We simulate the transfer characteristics of a scaled Hyper-FET constiting of a 22nm technology node FinFET (device characteristics simulated using the PTM[5] (Predictive technology model) in series with VO_2 having different channel dimensions (length and width; with thickness kept constant) as shown in Supplementary Figure 5a. Here, we consider the critical current density associated with the IMT and the MIT to be 10^5 A/cm^2 and 10^6 A/cm^2, respectively, as observed in our experimental data (Supplementary Figure 1b). The length and the width dimensions of the VO_2 are changed by the same ratio; this ensures that the resistance of the VO_2 device remains constant while the value of the critical currents (I_{IMT} and I_{MIT}) which depend only on the width (thickness constant) vary (I=J*t_{VO2}*W_{VO2}). Consequently, it can be observed from Supplementary Figure 5a that the hysteresis is modulated, and thus can be reduced. We also note that the enhanced trans-conductance (g_m) of the scaled conventional transistor also contributes to the reduced hysteresis. The minimum hysteresis (green) for the three cases studied here is calculated to be ~75 mV which is ~15% of the supply voltage (=0.5 V). Further, we also evaluate the response (transient) of a 3 stage inverter based on the Hyper-FET (Supplementary Figure 5b,c) with the smallest hysteresis (with L_{VO2}=8nm and W_{VO2}=16nm). The reduced hysteresis results in minimal effect on the rail-to-rail swing (Supplementary Figure 5c), thus enabling successful low-voltage inverter operation. Hence, optimizing the properties of the VO_2 in conjunction with the

properties of the MOSFET is crucial for harnessing the enhanced performance of the Hyper-FET device concept in circuit applications.

Supplementary References

1. Son, M. *et al.* Excellent Selector Characteristics of Nanoscale VO_2 for High-Density Bipolar ReRAM Applications. *IEEE Electron Device Lett.* **32,** 1579–1581 (2011).

2. Shukla, N. *et al.* Synchronized charge oscillations in correlated electron systems. *Sci. Rep.* **4,** 1–6 (2014).

3. Thathachary, A. *et al.* Investigation of $In_xGa_{1-x}As$ FinFET architecture with varying Indium (x) concentration and quantum confinement. T*ech. Digest Papers Symp. VLSI Technol*. 1-2 (IEEE, 2014).

4. Agrawal, A. *et al.* Enhancement Mode Strained (1.3%) Germanium Quantum Well FinFET (W_{Fin}=20nm) with High Mobility (Hole=700 cm^2/V.s), Low EOT (~0.7nm) on Bulk Silicon Substrate. *IEEE Int. Electron Device Meet.* 414-417 (IEEE, 2014).

5. Predictive Technology Model, http://ptm.asu.edu/, 2012.
